# Supplementary material for: Causal Relationship between Aging and Anorexia Nervosa: A White-Matter-Microstructure-Mediated Mendelian Randomization Analysis
Source: Biomedicines. 2024 Aug 16;12(8):1874. doi: 10.3390/biomedicines12081874 (PMC11351342; doi:10.3390/biomedicines12081874)
Supplement: Supplementary file 1 [file biomedicines-12-01874-s001.zip › Supplementary Figure/Figure S4/Singe SNP plot of MR analysis for TL to ubm-b-1881.pdf]

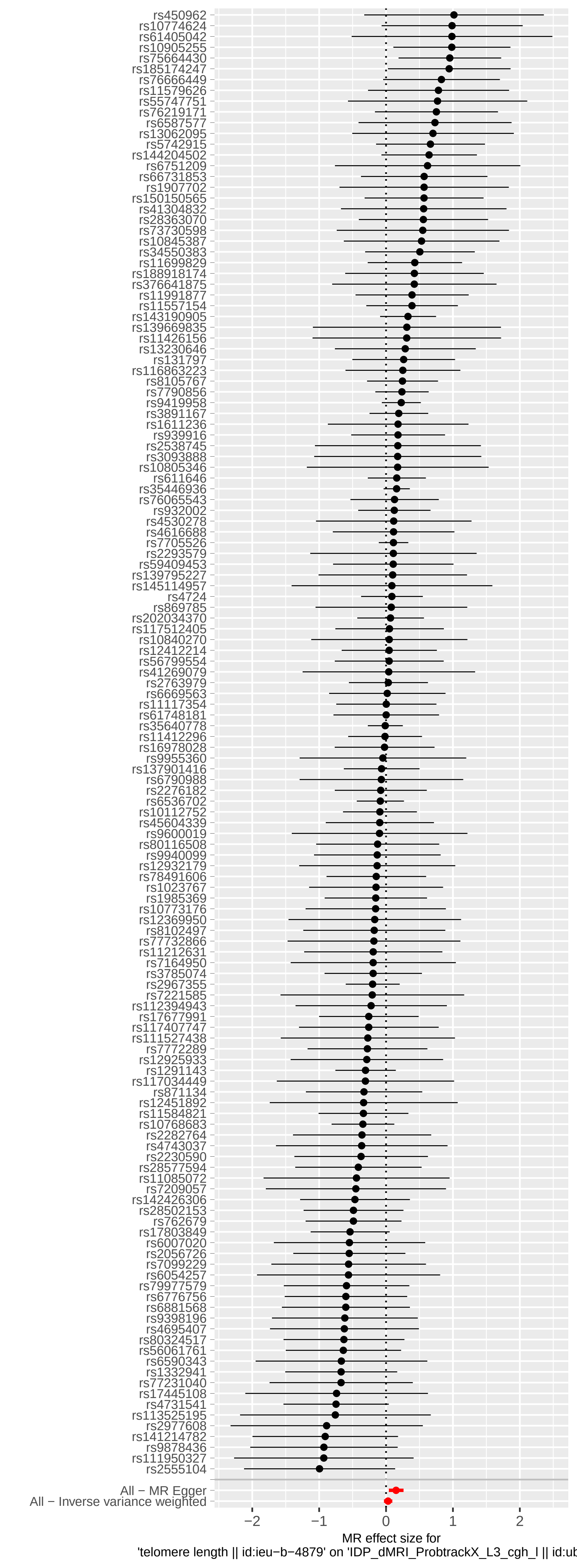
rs450962
rs10774624
rs61405042
rs10905255
rs75664430
rs185174247
rs76666449
rs11579626
rs55747751
rs76219171
rs6587577
rs13062095
rs5742915
rs144204502
rs6751209
rs66731853
rs1907702
rs150150565
rs41304832
rs28363070
rs73730598
rs10845387
rs34550383
rs11699829
rs188918174
rs376641875
rs11991877
rs11557154
rs143190905
rs139669835
rs11426156
rs13230646
rs131797
rs116863223
rs8105767
rs7790856
rs9419958
rs3891167
rs1611236
rs939916
rs2538745
rs3093888
rs10805346
rs611646
rs35446936
rs76065543
rs932002
rs4530278
rs4616688
rs7705526
rs2293579
rs59409453
rs139795227
rs145114957
rs4724
rs869785
rs202034370
rs117512405
rs10840270
rs12412214
rs56799554
rs41269079
rs2763979
rs6669563
rs11117354
rs61748181
rs35640778
rs11412296
rs16978028
rs9955360
rs137901416
rs6790988
rs2276182
rs6536702
rs10112752
rs45604339
rs9600019
rs80116508
rs9940099
rs12932179
rs78491606
rs1023767
rs1985369
rs10773176
rs12369950
rs8102497
rs77732866
rs11212631
rs7164950
rs3785074
rs2967355
rs7221585
rs112394943
rs17677991
rs117407747
rs111527438
rs7772289
rs12925933
rs1291143
rs117034449
rs871134
rs12451892
rs11584821
rs10768683
rs2282764
rs4743037
rs2230590
rs28577594
rs11085072
rs7209057
rs142426306
rs28502153
rs762679
rs17803849
rs6007020
rs2056726
rs7099229
rs6054257
rs79977579
rs6776756
rs6881568
rs9398196
rs4695407
rs80324517
rs56061761
rs6590343
rs1332941
rs77231040
rs17445108
rs4731541
rs113525195
rs2977608
rs141214782
rs9878436
rs111950327
rs2555104
All − MR Egger
All − Inverse variance weighted
−2
−1
0
1
2
MR effect size for
'telomere length || id:ieu−b−4879' on 'IDP_dMRI_ProbtrackX_L3_cgh_l || id:ub
